# Peer support for the maintenance of physical activity and health in cancer survivors: the PEER trial - a study protocol of a randomised controlled trial

**DOI:** 10.1186/s12885-019-5853-4

**Published:** 2019-07-03

**Authors:** Kirsten N. Adlard, David G. Jenkins, Chloe E. Salisbury, Kate A. Bolam, Sjaan R. Gomersall, Joanne F. Aitken, Suzanne K. Chambers, Jeff C. Dunn, Kerry S. Courneya, Tina L. Skinner

**Affiliations:** 1 School of Human Movement and Nutrition Sciences, The University of Queensland, Brisbane, QLD Australia; 2 Department of Neurobiology, Care Sciences and Society, Karolinska Institutet, Stockholm, Sweden; 3 School of Health and Rehabilitation Sciences, The University of Queensland, Brisbane, QLD Australia; 4 Cancer Council Queensland, Brisbane, QLD Australia; 5 Exercise Medicine Research Institute, Edith Cowan University, Perth, WA Australia; 6 Institute for Resilient Regions, University of Southern QLD, Springfield, QLD Australia; 7 Menzies Health Institute Queensland, Griffith University, Nathan, QLD Australia; 8 School of Public Health, The University of Queensland, Brisbane, QLD Australia; 9 Faculty of Health, University of Technology Sydney, Sydney, NSW Australia; 10 School of Social Science, The University of Queensland, Brisbane, QLD Australia; 11 Faculty of Kinesiology, Sport, and Recreation, University of Alberta, Edmonton, Alberta Canada

**Keywords:** Peer support, Oncology, Adherence, Exercise, Physical activity, Breast cancer, Colorectal cancer, Prostate cancer, Randomised controlled trial, Quality of life

## Abstract

**Background:**

Despite an overwhelming body of evidence showing the benefits of physical activity (PA) and exercise for cancer survivors, few survivors meet the exercise oncology guidelines. Moreover, initiating, let alone maintaining exercise programs with cancer survivors continues to have limited success. The aim of this trial is to evaluate the influence of peer support on moderate-to-vigorous PA (MVPA) and various markers of health 12 months following a brief supervised exercise intervention in cancer survivors.

**Methods:**

Men and women previously diagnosed with histologically-confirmed breast, colorectal or prostate cancer (*n* = 226), who are >1-month post-treatment, will be invited to participate in this trial. Once enrolled, participants will complete 4 weeks (12 sessions) of supervised high intensity interval training (HIIT). On completion of the supervised phase, both groups will be provided with written recommendations and verbally encouraged to achieve three HIIT sessions per week, or equivalent exercise that meets the exercise oncology guidelines. Participants will be randomly assigned to receive 12 months of peer support, or no peer support (control). Primary and secondary outcomes will be assessed at baseline, after the 4-week supervised HIIT phase and at 3-, 6- and 12-months. Primary outcomes will include accelerometry-derived MVPA and prescribed HIIT session adherence; whilst secondary outcomes will include cardiorespiratory fitness ($\dot{\mathrm{V}}{\mathrm{O}}_{2\mathrm{peak}}$), body composition, quality of life and select cytokines, myokines and inflammatory markers. Random effects mixed modelling will be used to compare mean changes in outcomes between groups at each time point. A group x time interaction will be used to formally test for differences between groups (alpha =0.05); utilising intention-to-treat analyses.

**Discussion:**

If successful, peer support may be proposed, adopted and implemented as a strategy to encourage cancer survivors to maintain exercise beyond the duration of a short-term, supervised intervention. A peer support-exercise model has the long-term potential to reduce comorbidities, improve physical and mental wellbeing, and significantly reduce the burden of disease in cancer survivors.

**Ethics:**

Human Research Ethics Committee of Bellberry Ltd. (#2015–12-840).

**Trial registration:**

Australian New Zealand Clinical Trial Registry 12618001855213. Retrospectively registered 14 November 2018.

Trial registration includes all components of the WHO Trial Registration Data Set, as recommended by the ICMJE.

## Strengths and limitations of this trial

- This is the first randomised controlled trial to investigate the effectiveness of peer support for maintaining physical activity following a brief supervised training intervention in cancer survivors.
- This trial will monitor changes in physical activity, physical fitness, body composition, blood markers of health, and indicators of quality of life for 12-months.
- If peer support is found to improve maintenance of exercise for 12 months following a brief supervised training intervention, it will emerge as an effective means to improve the health and wellbeing of people living with and beyond cancer.
- The applications of this trial will be limited to people living with and beyond breast, colon and/or prostate cancer.

## Background

Cancer incidence is rapidly increasing with an estimated 18.1 million new cases and 9.6 million cancer deaths worldwide in 2018 [1]. Whilst two thirds of all people diagnosed with cancer survive at least 5 years, many live with reduced functional capacity, poor health-related quality of life and high levels of fatigue [2, 3]. Clinically significant cancer treatment-related adverse effects can last for up to 10 years post-diagnosis; the enduring nature of such effects highlights the need for long-term management strategies to improve outcomes following diagnosis and treatment for people with cancer [4, 5].

Higher levels of physical activity (PA) is associated with a reduced risk of cancer recurrence [6], with an estimated 30–40% risk reduction for both breast and colorectal cancer survivors [7, 8]. Emerging and accumulating evidence also shows that PA may reduce the risk of prostate cancer recurrence [9]. Moreover, exercise interventions for cancer patients and survivors has also been shown to reduce cancer-related fatigue [10], and improve psychological wellbeing and quality of life [11], physical function [12], body weight and composition [13], muscle strength and endurance [12], immune function [14], and cardiorespiratory fitness [15].

A growing body of research shows that relatively high intensity exercise can elicit rapid and significant improvements in various markers of health across a range of different clinical populations [16–18]. Devin et al. (2016) recently showed that 4 weeks of high intensity interval training (HIIT) significantly increased cardiorespiratory fitness ($\dot{\mathrm{V}}{\mathrm{O}}_{2\mathrm{peak}}$, + 3.5 mL.kg^− 1^.min^− 1^; *p* < 0.001) and led to significant decreases in fat mass (− 0.74 kg; *p* < 0.002) and increases in lean mass (+ 0.72 kg; *p* < 0.001) in colorectal cancer survivors [19]. These results are clinically meaningful when placed in the context of the Cooper Centre Longitudinal Study results which estimated that an increase in $\dot{\mathrm{V}}{\mathrm{O}}_{2\mathrm{peak}}$ of 3.5 mL.kg^− 1^.min^− 1^ was associated with a 10% (hazard ratio, 0.90 95% confidence interval [CI] [0.84–0.97]) decrease in cancer-specific mortality risk [20]. Importantly, three HIIT sessions per week, each comprising only 16 min of high intensity exercise interspersed with active recovery, allowed participants to meet the ‘cardiovascular’ component of the current exercise oncology guidelines (150 min per week of moderate or 75 min per week of vigorous aerobic exercise or an equivalent combination) [21, 22]. In addition to HIIT being a time-efficient, highly effective means of rapidly improving health, Kampshoff et al. [23] have recently reported that high intensity exercise, when compared to low-to-moderate intensity exercise, results in greater improvements in quality of life. When these findings are considered with those of Martin et al. [24], who found that higher intensity exercise helps cancer survivors maintain their motivation to exercise, the available, emerging research indicates that HIIT holds potential for addressing the long-term health of cancer survivors.

Despite the strong body of evidence showing the benefits of PA and exercise for cancer survivors, research suggests that very few cancer survivors meet the exercise oncology guidelines [25–27]. Indeed, some studies have shown that cancer survivors are less active than those who have not had cancer [28]. For example, Galvao et al. [26] reported only 12% of prostate cancer survivors meet the current exercise oncology guidelines [21]. In addition, Gomersall et al. [29] found that less than 25% of participants who start a physical activity program remain active after 6 months [30]. Similarly, Courneya et al. [31] found that without support, only 36% of patients with lymphoma met exercise oncology guidelines 6 months following an intervention. These results substantiate the colloquially-known ‘drop-off’ in PA participation following the completion of PA interventions and may explain the typically poor maintenance of health outcomes at longer-term follow-up assessments post-intervention.

Maintaining exercise adherence and its subsequent health benefits following supervised exercise interventions is therefore an area of particular need in exercise oncology [2, 32]. To date, variations of support for improving and maintaining the activity levels of cancer survivors have included phone, text reminders and printed materials [33]. Although these methods of behavioural support have shown efficacy in cancer populations in the short to mid-term [34, 35], they also have some inherent limitations, with the most prominent being a lack of face-to-face interaction.

Peer support, which is underpinned by components of social cognitive theory [36] and the theory of planned behaviour [37], uses trained individuals who have shared experiences and who provide knowledge, emotional, social and/or practical help to support others. Though peer support has been used to maintain physical activity levels in patients recovering from cardiac surgery [38], its effectiveness as a means of promoting and/or maintaining physical activity in cancer survivors following a brief supervised intervention warrants further investigation [39]. To date, interventions have seen promising increases in PA among peer-supported cancer survivors compared to contact controls [40, 41]. However, these trials were conducted solely with female breast cancer survivors. Thus, the generalizability of the findings to survivors of other cancers and male cancer survivors remains unknown. In addition, these interventions were solely telephone-based despite interactive mediums of intervention delivery (e.g. face-to-face delivery) being particularly important for people in the early stages of behaviour change [42]. Furthermore, these interventions were relatively short, with support lasting only 12 weeks. Therefore, the durability of these findings remains unknown.

The primary aim of this trial is to evaluate the influence of peer support compared to no peer support on MVPA 12 months following a brief supervised exercise intervention in cancer survivors. Secondarily, this trial aims to determine the influence of peer support on various exercise-related markers of health (cardiorespiratory fitness, body composition, strength, quality of life, blood biomarkers, overall physical activity and sedentary behaviour) 12 months following a brief supervised exercise intervention in cancer survivors compared to those without peer support. It is hypothesised that peer support will result in higher levels of MVPA and markers of health 12 months follow a brief supervised exercise intervention compared to those without peer support.

## Methods

### Study design

This two-arm randomised controlled trial will involve men or women with a previous diagnosis of breast, colorectal or prostate cancer. All participants and peer supporters will first complete 4 weeks (12 sessions) of HIIT supervised by Accredited Exercise Physiologists (AEP) at the School of Human Movement and Nutrition Sciences, The University of Queensland, Brisbane, Australia (‘supervised phase’). On completion of the supervised phase, participants will be stratified by gender and age before being randomly assigned (1:1) to either a peer support group or a non-peer support group. All will be provided with written recommendations to maintain three HIIT sessions per week, or an alternative equivalent that meets the exercise oncology guidelines (i.e. 150 min per week of moderate or 75 min per week of vigorous aerobic exercise or an equivalent combination), for 12 months [21, 22]. Those assigned to the peer support group will have access to a peer who will provide support to meet the HIIT and/or exercise guidelines.

**Fig. 1:**
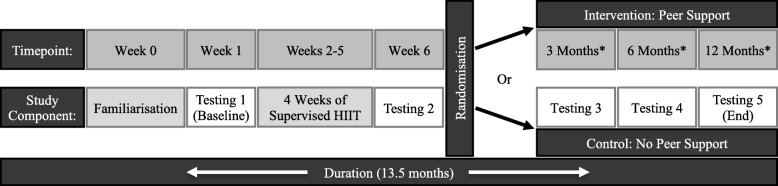
Schematic timeline of the study. *Key:* *: following randomisation; HIIT: high intensity interval training

All participants will complete a series of assessments (see Table 1) at baseline, at the end of the supervised phase, and at 3-, 6- and 12-months following the supervised phase (see Fig. 1).

**Table 1:** Schedule of data collection

Assessment or Outcome	Study Period						
*Time Point:*	*Pre-FM*	*FM*	*Pre-SP*	*Post-SP*	*3 M*	*6 M*	*12 M*
*Screening*							
Participant eligibility	✓						
Adult Pre-Exercise Screening System	✓						
Informed consent	✓						
Doctor’s consent	✓						
Medical history and current status	✓	✓	✓	✓	✓	✓	✓
Demographics	✓						
Concomitant research study participation	✓	✓	✓	✓	✓	✓	✓
*Primary Outcomes*							
Moderate-to-vigorous physical activity (MVPA)			✓	✓	✓	✓	✓
*Secondary Outcomes*							
Cardiorespiratory fitness ($\dot{\mathrm{V}}{\mathrm{O}}_{2\mathrm{peak}}$)		✓	✓	✓	✓	✓	✓
Body composition (DXA)			✓	✓	✓	✓	✓
Anthropometry (BMI)			✓	✓	✓	✓	✓
Strength (isometric grip strength)			✓	✓	✓	✓	✓
Blood biomarkers (IL-6, IL-8, Hepcidin, TNF-α, IGF-1, IGF-2, IGFBP-3 and CRP)			✓	✓	✓	✓	✓
Self-reported physical activity (Godin)			✓	✓	✓	✓	✓
Sedentary behaviour and light physical activity (accelerometry)			✓	✓	✓	✓	✓
Quality of life and wellbeing (EORTC QLQ C-30, SF-36 and FACT-G)			✓	✓	✓	✓	✓
Cancer-related fatigue (FACIT-F)			✓	✓	✓	✓	✓
Insomnia (ISI)			✓	✓	✓	✓	✓
Symptom distress (BSI)			✓	✓	✓	✓	✓
Depression and anxiety (DASS, PHQ-9 and GAD-7)			✓	✓	✓	✓	✓
Participant:peer supporter relationship (Therapeutic Alliance)			✓	✓	✓	✓	✓
Self-efficacy			✓	✓	✓	✓	✓
Barriers and enablers to PA			✓	✓	✓	✓	✓
Tertiary Outcomes							
Semi-structured interviews							✓
Physical activity preferences			✓	✓	✓	✓	✓
Economic evaluation (AQoL-8D)			✓	✓	✓	✓	✓
Adverse and serious adverse events		✓	✓	✓	✓	✓	✓

*Key: Pre-FM* pre-familiarisation, *FM* familiarisation, *Pre-SP* pre-supervised phase, *Post-SP* post-supervised phase, *3 M* 3-months post-randomisation testing, *6 M* 6-months post-randomisation testing, *12 M* 12-months post-randomisation testing, $\dot{\mathrm{V}}{\mathrm{O}}_{2\mathrm{peak}}$ peak volume of oxygen consumption, *DXA* dual energy X-ray absorptiometry, *BMI* body mass index, *IL-6* interleukin 6, *IL-8* interleukin 8, *TNF-α* tumour necrosis factor – alpha, *IGF-1* insulin-like growth factor 1, *IGF-2* insulin-like growth factor 2, *IGFBP-3* insulin-like growth factor-binding protein 3, *CRP* C-reactive protein, *Godin* Godin Leisure-Time Physical Activity Questionnaire, *EORTC QLQ C-30* European Organization for Research and Treatment of Cancer quality of life questionnaire for people with cancer, *SF-36* Short form 36 health survey, *FACT-G* Functional Assessment of Cancer Therapy: General, *FACIT-F* The Functional Assessment of Chronic Illness Therapy: Fatigue, *ISI* Insomnia Severity Index, *BSI* Brief Symptom Inventory, *DASS* Depression Anxiety Stress Scale, *PHQ-9* Patient Health Questionnaire-9, *GAD-7* Generalised Anxiety Disorder 7-item scale, *PA* physical activity, *AQoL-8D* Assessment of Quality of Life – 8 Dimensions Multi-Attribute Utility Instrument

### Participants

Men and women previously diagnosed with histologically-confirmed breast, colorectal or prostate cancer (*n* = 226) will be invited to participate.

#### Inclusion criteria

i. ≥ 18 years old;
ii. ≥ 1-month post-treatment for cancer and not anticipating undergoing further treatment during the study period; and
iii. Able to travel to the exercise testing and training premises.

#### Exclusion criteria

i. Currently performing any regular HIIT sessions (> 4 sessions in the previous month);
ii. The presence of musculoskeletal, neurological, respiratory, metabolic or cardiovascular conditions that may prevent safe completion of the exercise demands of the study; and
iii. Participant is pregnant or anticipates becoming pregnant during the study period.

Participants will be required to obtain their doctor’s approval and will be individually screened using a medical history form and interview to confirm eligibility.

#### Discontinuation criteria

Individual participants will be discontinued from the trial, in agreement with the participant’s doctor where appropriate, if:

i. The participant is diagnosed with recurrence of cancer and needs to recommence active cancer treatment;
ii. It is deemed unsafe for the participant to continue exercising without specialist exercise professional supervision during the unsupervised phase of the trial;
iii. The participant has any major surgery or health condition arise within the study period that will significantly affect their ability to participate in exercise for a duration > 1 month;
iv. The participant becomes pregnant; or
v. The participant elects to withdraw their consent for any reason.

### Recruitment

#### Participant recruitment

Participants will be recruited from the University of Queensland, by way of media releases, presentations, advertisements in newsletters, newspapers, social media and noticeboards. Participants will also be recruited via the Cancer Council Queensland (CCQ) support group network, CCQ 131120 telephone support service, CCQ Registries and the Queensland Health Cancer Registry. Potential participants, after meeting the eligibility criteria and being advised of the study requirements by the principal investigator, will receive the Participant Information Sheet, Participant Consent Form, Medical History Form and Medical Doctor Consent Form. Each will then be contacted by telephone by the principal investigator to discuss any questions they may have regarding the trial. If they wish to continue they will be required to obtain their Doctor’s written consent and sign the Participant Consent Form, both of which will be collected and securely stored by the primary investigator.

#### Recruitment and training of peer supporters

Peer supporters, who will also be cancer survivors, will be recruited through CCQ’s existing peer-support network in addition to the aforementioned participant recruitment pathways. Peer supporters will complete the same consent and screening procedures as the study participants. Each peer supporter will undergo CCQ’s peer support training course, delivered over one or two consecutive days (approximately 8 h per day). CCQ’s course helps to equip volunteers with the skills required to provide appropriate emotional support to those affected by cancer, based on their shared experience with cancer either as a patient; the premise of which originates from the Social Cognitive Theory [36]. CCQ’s peer support training is also an opportunity for prospective peer supporters to develop a better understanding of their upcoming role, to ask questions, and to hear from more established volunteers. Course outcomes aim to educate peer supporters on how to support their participant to build motivation, improve confidence and facilitate self-efficacy within the exercising context.

The CCQ peer support training is primarily delivered face-to-face with an accompanying pre-reading package outlining CCQ values, organisational structure, and introductory knowledge of cancer, treatments, and CCQ support services. Information delivered face-to-face utilizes digital presentations, scenario group work, and personal reflections. Moreover, didactics and role play with investigator feedback will be used to enhance the peer supporters’ counselling skills. Peers will also receive training on monitoring participant safety during exercise and what to do should an adverse or serious adverse event occur. An adverse event will be defined as ‘any untoward medical occurrence in a participant subject to the intervention’. A severe adverse event will be defined as ‘any event requiring hospitalisation or causing an inability to carry out usual activities’. Adverse events will be assessed by monitoring and recording during all exercise sessions by the supervising Exercise Physiologist.

Peer supporters will perform the same supervised 4-week HIIT phase as described for the participants to familiarise them with the demands of the prescribed exercise protocol. The supervising AEP uses these sessions to further educate and reiterate to the peer supporters on the specifics of the exercise protocols and safety measures.

### Randomisation

Following completion of the 4-week supervised training phase and the second testing session, participants will be stratified by sex and age before being randomly assigned (1:1 allocation ratio) to either a peer support group or a non-peer support group. Randomisation will be performed using a computerised random number generator by a research officer independent to the study. Non-systematic block sizes of four, six, or eight will be used to ensure allocation concealment. Randomisation history will be kept secure by the independent research officer in an electronic password-protected file. The independent research officer will confidentially communicate the allocation result to the trial’s primary investigator within 48 h after completing outcome testing following the second testing session. All investigators will be blinded to group allocation for baseline testing, the supervised phase and for testing following the supervised phase, with randomisation occurring after the completion of testing following the supervised phase. The primary outcome will be analysed by a researcher blinded to group allocation. Unblinding will not be permissible under any circumstances. A schematic representation of the study protocol is shown in Fig. 1.

### 4-week supervised high intensity interval training phase

**Fig. 2:**
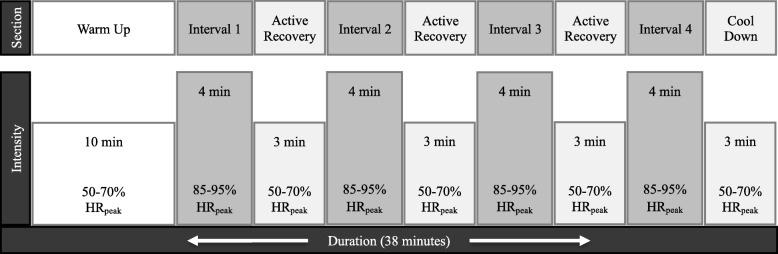
Schematic representation of the HIIT protocol. *Key:* HR_peak_: Peak heart rate, as determined by the most recent peak oxygen consumption ($\dot{\mathrm{V}}{\mathrm{O}}_{2\mathrm{peak}}$) test; min: minutes

Following familiarisation with the procedures, participants will complete 12 HIIT sessions over 4 weeks, supervised by an AEP. Training sessions will be conducted at the School of Human Movement and Nutrition Sciences, The University of Queensland, and be performed in small groups of less than five participants. Prior to exercising, participants’ heart rate and blood pressure will be measured to indicate any contraindications to commencing exercise as outlined by the American College of Sports Medicine [43]. Each training session will follow the procedure as previously described by Devin et al. [19] (see Fig. 2).

### 12-month maintenance phase

Following completion of the 4-week supervised phase, all participants will be encouraged to maintain three HIIT sessions a week – a training commitment that will have them meet the ‘cardiovascular’ component of the American College of Sports Medicine’s exercise oncology guidelines. All participants will be provided with 12-months free access to exercise training facilities located at multiple sites across South-East Queensland. Each exercise facility will be fitted with specific cycle ergometers and heart rate monitors to enable participants to complete HIIT sessions, and for the research staff to accurately monitor all sessions over the 12-month maintenance phase.

### Peer support intervention

Participants randomised to the peer support group will be assigned a peer supporter. Assignment will be determined by training time availability and preference to enhance the ease of scheduling face-to-face exercise sessions. The peer supporter will maintain weekly contact (by phone, email and/or joining training sessions) with their participant for the 12-month maintenance phase. Contact frequency and type will be diarised by the peer supporter following each occurrence. The peer supporter’s goal will be to encourage their participant to maintain physical activity levels throughout the 12-month training period, equivalent to those achieved during the supervised phase. Peer supporters will be encouraged to use the knowledge and strategies gained from the CCQ peer support training sessions during discussions with their participants i.e. goal setting and planning, facilitating self-efficacy, improving confidence, overcoming barriers and building motivation. Where peer supporters have difficulty maintaining the recommended weekly contact regime, they will be encouraged to notify the research team to discuss and apply appropriate strategies moving forward. Participants will be required to diarise each exercise training session. Cycle ergometers will record each exercise session and data will be periodically retrieved by the researcher team. The training diaries and cycle ergometer data, in conjunction with the accelerometer data, will be used to assess maintenance to physical activity and the prescribed exercise over the 12 months.

### Outcomes

Primary and secondary outcomes will be measured at baseline, at the end of the 4-week supervised phase, and at 3-, 6- and 12-months following the supervised phase. See Table 1 for a visual representation of the data collection schedule.

Assessments will be performed in a consistent order to allow for appropriate rest periods and to ensure that successive outcomes are minimally influenced by accumulative testing-fatigue. Participants will be advised to take prescribed medications as normal throughout the study period. Concomitant study participation will be documented at each testing session and the primary investigator will determine whether any co-intervention bias may influence the trial outcomes. Hospital admissions and any alterations in medical status or medications will be recorded at each assessment for future reference during analysis of results.

#### Testing standardisation measures

At each testing session, participants will be asked to: (i) maintain a hydrated state in the 24 h prior to testing; (ii) abstain from caffeine and alcohol intake for 12 h prior to testing; and (iii) avoid any vigorous, high or unaccustomed moderate intensity exercise or physical activity for the preceding 48 h. Prior to the baseline testing session, participants will be required to record a 3-day diet diary, of which they will follow prior to all subsequent testing sessions. Participant adherence to the testing standardisation measures will be evaluated prior to the commencement of each assessment. If testing standardisation measures are not met, the assessment will be rescheduled for the following day and the leading researcher will reinforce the participant’s understanding of the pre-testing requirements.

### Primary outcome

#### Moderate-to-vigorous physical activity

The primary outcome for this study will be MVPA at 12 months. MVPA can be accrued through performance of the prescribed HIIT sessions and/or by engaging in any MVPA. Daily physical activity will be assessed through the use of training diaries, retrieval of HR and Wattbike ergometer data from each training session, as well as Actigraph GT3X+ accelerometry (Pensacola, Florida). Participants will be asked to wear an accelerometer on an elastic waist belt aligned with the right anterior axillary line for 7 days immediately preceding baseline, 3-, 6- and 12-month assessments. Participants will be asked to complete a brief log to record when they wear the monitor, including wake and sleep times and if they removed the monitor for > 10 min. They will be instructed to wear the device at all times, excluding sleep, any water-based activities (e.g. showering/swimming) and contact sports. Accelerometers will be initialised with 30 s epochs and a 30 Hz sampling frequency. A valid day will be defined as a minimum wear-time of 10 waking hours, with non-wear time defined as 60 min or more of consecutive activity counts of zero. For activity data to be included, participants must satisfy a minimum wear-time criterion of at least four of the 7 days, with at least one of the days being a weekend day [44, 45]. Minutes of MVPA will be calculated using Actilife v6 (Pensacola, Florida) and the cutpoints described by Troiano et al. [44].

### Secondary outcomes

#### Cardiorespiratory fitness ($\dot{\mathrm{V}}{\mathrm{O}}_{2\mathrm{peak}}$)

Cardiorespiratory fitness represents a strong predictor of decreased total cancer mortality risk, independent of adiposity [46, 47]. $\dot{\mathrm{V}}{\mathrm{O}}_{2\mathrm{peak}}$ and exercise duration during the $\dot{\mathrm{V}}{\mathrm{O}}_{2\mathrm{peak}}$ test will be used as measures of cardiorespiratory capacity. The $\dot{\mathrm{V}}{\mathrm{O}}_{2\mathrm{peak}}$ test will be conducted as previously described by Devin et al. [48].

#### Body composition

High adipose tissue and reduced skeletal muscle has been associated with poorer short-term recovery following cancer treatment and poorer survival [49, 50]. Furthermore, a decline in bone mineral density, particularly trabecular density, has been correlated with increased fracture risk and subsequent decreased quality of life [51]. Regional and whole-body lean mass, fat mass and bone mineral density will be measured using dual energy x-ray absorptiometry (DXA, Hologic Discovery A, Waltham, MA) as previously described by Devin et al. [19] and Bolam et al. [52]. The coefficients of variation values in our laboratory are < 1.1%.

#### Anthropometry

Obesity, particularly around the abdomen, has been directly associated with risk of several types of cancer, including cancer of the breast and colon. In addition, both body mass index (BMI) and waist circumference have been separately associated with a decrease in global health status, functioning and symptoms of fatigue [53]. Therefore, prevention of weight gain is important for reducing the risk of cancer recurrence [54–56].

Height and body mass will be measured using a stadiometer (Seca, Birmingham, UK) and electronic scales (A & D Mercury, Pty Ltd., Thebarton, Aus.), respectively, from which BMI will be calculated. Waist and hip circumference will be measured using an anthropometric steel tape measure (Lufkin W606 M retractable steel tape; Cooper Tools), from which a waist-to-hip ratio will be determined. All anthropometrics will be measured according to the International Society for the Advancement of Kinathropometry (ISAK) procedures [57]. Measures will be recorded to the nearest 0.1 cm or 0.01 kg in duplicate, with mean values used in subsequent analyses. If duplicate measures vary by ≥2%, a third measure will be taken with the median value used in subsequent analyses.

#### Isometric grip strength

Cancer treatment commonly results in reduced muscle strength which leads to decreased functional capacity and potential declines in quality of life and survival [58, 59]. Dominant and non-dominant handgrip strength will be assessed using a spring-loaded grip dynamometer (TTM, Tokyo, Japan) to estimate physical performance and muscular strength [60]. Participants will perform a 3-s maximal contraction with each hand maintaining a 90^o^ elbow flexion and limit accessory movements [61]. Three trials will be performed on each side, alternating between the dominant and non-dominant hand with the best result used in subsequent analysis. A rest period of at least 30 s will be provided between subsequent attempts.

#### Blood biomarkers

Resting samples of venous blood (20 ml) will be drawn by a qualified and experienced phlebotomist. Plasma and serum samples will be separated according to standard procedures and stored in a − 80 °C freezer for subsequent batch testing. Plasma samples will be analysed for interleukin 6, interleukin 8, tumour necrosis factor – alpha and C-reactive protein, which have been identified as key mediators of neuro-immune interactions associated with post-treatment fatigue in cancer survivors [62, 63]. Additional analyses of insulin-like growth factor-1 (IGF-1), − 2 (IGF-2), and -binding protein-3 (IGFBP-3) will be explored as plausible mechanisms underpinning the inverse association between physical activity and cancer progression [64]. Plasma cytokines will be assessed using high-sensitivity ELISAs (R&D Systems, Minneapolis, MN) following the manufacturer’s specifications. Quality control procedures for the laboratory will be conducted in the manner reported by Aziz et al. [65]. All samples for a given participant will be run in parallel to minimise inter-assay variability.

#### Self-reported physical activity (Godin leisure-time physical activity questionnaire)

Leisure-time PA is a primary domain of intervention and research in public health [66]. Compared to other forms of physical activity (e.g. occupational), leisure-time PA may provide the greatest opportunity for enjoyment and improvement in cardiorespiratory fitness and health [67, 68]. The Godin Leisure-Time Physical Activity Questionnaire is a frequently used measure of leisure-time PA and will be completed at all testing time points [69]. The questionnaire requires participants to recall during a typical 7-day week the frequency and duration of exercise completed at three separate intensities: mild, moderate, and strenuous intensity. These values are used to calculate a weekly leisure-time activity index based on summing the frequency of sessions completed multiplied by a weighting factor for each level of intensity. The Godin questionnaire has been shown to have a modest correlation compared with accelerometry-derived measures of physical activity (r = 0.45) but demonstrates high test-retest reliability (r = 0.75) [70–72].

#### Sedentary behaviour and light physical activity

Sedentary behaviour (SB) is an independent risk factor for cancer incidence and mortality [73]. Comparably, moderate-to-vigorous intensity exercise generally delivers the greatest health benefits [74]. Exercise guidelines for people with cancer [22], however, acknowledge that any PA, including light physical activity (LPA), is better than none [73, 75]. Therefore, SB and LPA will be assessed using Actigraph GT3X+ accelerometry (Pensacola, Florida), following the methods described for MVPA. Time spent in SB will be calculated using Actilife v6 (Pensacola, Florida) and the cutpoints described by Matthews et al. [45] and time spent in LPA will be determined as the time spent above the SB cutpoint (≤ 100 counts per minute) and below the MVPA cut-point (≥ 2020 counts per minute) [44, 45].

#### Cancer-specific quality of life (EORTC QLQ-C30)

The European Organization for Research and Treatment of Cancer quality of life questionnaire for people with cancer is designed to assess quality of life in cancer patients and holds good internal consistency and clinical validity [76]. The EORTC QLQ-C30 [76–79] is a 30-item cancer-specific instrument including nine multi-item scales; five functional scales (physical, role, cognitive, emotional, and social); three symptom scales (fatigue, pain, and nausea and vomiting); and a global health and quality-of-life scale. The EORTC QLQ-C30 questionnaire has been shown as reliable and valid for use in cancer populations [78].

#### General health and wellbeing (SF-36)

The Medical Outcomes Study 36-item short-form 36 Health Survey (SF-36) will be used to assess general health and wellbeing domains of physical and mental health [80]. The SF-36 is scored through a set of eight scales encompassing physical and mental health measures. This survey has been shown to be reliable and has been validated for comparing general and specific populations, and estimating the relative burden of disease [81].

#### Health-related quality of life (FACT-G)

Quality of life will be measured using the Functional Assessment of Cancer Therapy-General (FACT-G) self-report questionnaire [82]. The FACT-G comprises four subscales: Physical Well-Being (PWB), Social/Family Well-Being (SWB), Emotional Well-Being (EWB), and Functional Well-Being (FWB). The FACT-G has been widely used in clinical trials, is relatively simple, and has demonstrated sensitivity according to the performance status and extent of disease [82, 83]. This questionnaire has been extensively used in oncology clinical trials, due to its ease of administration, brevity, reliability, validity, and responsiveness to clinical change [82]. The English language version of the FACT-G will be self-administered by participants.

#### Cancer-related fatigue (FACIT-F)

Fatigue will be assessed by the Functional Assessment of Chronic Illness Therapy Fatigue Subscale Questionnaire (FACIT-F) as described by Yellen et al. [84]. The FACIT-F is a 13-item fatigue subscale utilising a five-point Likert self-report scale ranging from 0 (not at all) to 4 (very much so). The total score varies from 0 (worst condition) to 52 (best condition) and is calculated as a sum of all items. The score is determined after re-parameterization of items 7 (I have energy) and 8 (I am able to do my usual activities) where 0 is worst condition and 4 is best condition, which have an inverse relationship to the other 11 subscale items. The FACIT-F questionnaire is reliable and has previously been validated for predicting efficacy in treatment outcomes in oncological-induced fatigue with high sensitivity (0.92) and reasonable specificity (0.69) [85]. Furthermore, convergent and discriminant validity testing has previously demonstrated a significant positive relationship with other measures of fatigue and a negative correlation with vigour [86].

#### Insomnia (ISI)

The Insomnia Severity Index (ISI) is a seven-item measure of the nature, severity and impact of insomnia on a Likert scale (0 = *not at all*, to 4 = *very much*) [87]. Questions relate to the subjective qualities of the respondents’ sleep, including satisfaction with sleep patterns, the degree to which insomnia interferes with daily functioning, and how the respondent feels their insomnia is noticeable to others [88]. The ISI has shown internal validity (r = 0.76) in insomnia diagnosis [87], and reliability of α = 0.90 in breast and prostate cancer [89].

#### Psychological distress (BSI)

The Brief Symptom Inventory (BSI) [90] is a 53-item self-report symptom inventory that assesses nine patterns of clinically relevant psychological symptoms. It is a brief version of the Symptom Checklist List 90-R (SCL-90-R). Correlations between the BSI and SCL-R-90 are reported to range from 0.92 to 0.99 [90, 91]. Participants rate the extent to which they have been bothered (0 =“not at all” to 4 = “extremely”) in the past week by various symptoms [91]. The nine dimensions assessed are: somatization, obsession-compulsion, interpersonal sensitivity, depression, anxiety, hostility, phobic anxiety, paranoid ideation and psychoticism. The BSI also includes three indices of global distress: Global Severity Index, Positive Symptom Distress Index, and Positive Symptom Total. The BSI has been used in a variety of clinical and counselling settings as a mental health-screening tool and as a method of measuring symptom reduction during and after treatment. Research has previously found somatisation and anxiety to be related to physical activity levels in colorectal cancer survivors [92].

#### Depression, anxiety and stress scale (DASS)

The DASS questionnaire is a 42-item self-report instrument design to measure the three related negative emotional states of depression, anxiety, and stress [93]. Participants will be asked to plot their relative emotional feelings on a 0–3 scale (i.e. 0 being ‘did not apply to me at all’ and 3 being ‘applied to me very much, or most of the time) over each emotional domain [93]. The DASS has been clinically validated in measuring emotional states of depression, anxiety and stress in clinical populations and has good reliability and moderate sensitivity [94].

#### Depression and generalised anxiety (PHQ-9)

Depression is highly prevalent among people with cancer, with rates of depressive spectrum disorders as high as 58% [95]. The Patient Health Questionnaire-9 (PHQ-9) is a multipurpose instrument that will be used to assess severity of depression [96]. The diagnostic validity of the PHQ-9 was established in studies involving eight primary care and seven obstetrical clinics. PHQ scores ≥10 had a sensitivity of 88% and a specificity of 88% for major depression [96, 97]. PHQ-9 scores of 5, 10, 15, and 20 represents mild, moderate, moderately severe and severe depression [98].

#### Generalised anxiety disorder (GAD-7)

Approximately 20% of long-term cancer survivors report anxiety symptoms [99]. The GAD-7 is a self-administered patient questionnaire used as a screening tool and severity measure for generalised anxiety disorder [100]. GAD-7 has seven items, which measure severity of various signs of GAD according to reported response categories with assigned points [100].

#### Participant:peer supporter relationship

The quality of the bond between the peer-supporters and their participants will be assessed using an adapted version of the Working Alliance Inventory (WAI) questionnaire [101]. The WAI is a 7-point Likert-type measure that assesses 12 alliance dimensions including ‘agreement on task’, ‘agreement of goals’ and ‘development of bonds’, amongst others [102, 103]. The WAI has demonstrated high construct validity and high internal consistency estimates [103]. The WAI has been adapted for terminology relevance regarding ‘HIIT’ and ‘peers’ in this study. Furthermore, the 7-point Likert scale has been condensed to a 5-point Likert scale for consistency and ease of interpretation with the other study questionnaires.

#### Self-efficacy

To assess self-efficacy beliefs, participants will review the exercise recommendations for this trial (3 x HIIT sessions per week), and indicate the extent to which they feel capable of successfully adhering to these recommendations. Specifically, participants will be asked to what extent they would rate the truth of the statement ‘I feel capable of successfully completing 3 x HIIT sessions per week for 4 weeks, 3 months, 6 months, 9 months, and 12 months’, respectively. Participants will be asked to rate their responses on a 5-point Likert scale ranging from ‘strongly disagree’ to ‘strongly agree’. This method of assessing self-efficacy has been adapted from the Self-Efficacy for Exercise (SEE) scale which was originally described and validated in older adults by Resnick & Jenkins [104].

#### Barriers and enablers to exercise participation

Barriers and enablers to exercise participation will be assessed via two open-ended written questions about barriers and enablers to physical activity/exercise, as described by Evenson et al. and Macniven et al. [105, 106]. The barrier question will ask: ‘What is the one main reason that makes it harder for you to be more active?’. The enabler question will ask: ‘What is the one main reason that helps you to be more active?’. Two investigators will code responses and any discrepancies will be resolved by consensus. These items will be grouped into meaningful categories considering the socioecological framework, with intrapersonal, interpersonal, neighborhood or environmental, and organizational or policy categories.

Participants will also be asked to provide statements based on the Behavioural Regulation in Exercise Questionnaire Version 2, as previously used by Martin et al. and Markland et al. [24] [107]. Participants will be asked to respond to matrix-based questions such as ‘I find it difficult to exercise because of fatigue’ and ‘I am motivated to perform exercise because it will improve my physical health’. Participants will rate their agreement with the statements on a 5-point Likert scale ranging from ‘strongly disagree’ to ‘strongly agree’.

### Tertiary outcomes

#### Semi-structured interviews

At the end of each individual’s involvement in the study, participants and peer supporters will be invited to partake in a semi-structured interview (approximately 1 h) in-person or via telephone regarding their views and experiences throughout the trial. This will include study completers, as well as those who withdraw or fail to complete the study. Interview questions will focus on the participant’s experiences as a cancer survivor, with exercise and their participation in the trial. Peer supporters and participants who were randomised to receive peer support will be asked additional questions on their experiences providing and receiving peer support, respectively. Interviews will be audio-recorded and transcribed verbatim before undergoing inductive thematic analysis [108].

#### Physical activity preferences

The physical activity preference questionnaire will be used to assess the physical activity context preferred by participants throughout the duration of the trial due to its potential role as a covariate [109]. The questionnaire assesses physical activity preferences for 14 contexts of format (e.g. vigorous), location (e.g. outdoors), and social setting (e.g. done alone). Participants will be asked to provide ratings that reflect their agreement with one of two bi-polar statements at each end of the continuum (e.g. ‘Strongly disagree’ – ‘Strongly agree’) [110].

#### Assessment of quality of life – 8 dimensions multi-attribute utility instrument (AQoL-8D)

The AQoL-8D is a Multi-Attribute Utility (MAU) instrument which will be used to assess health state utility at baseline, immediately following the supervised phase, and at 3, 6 and 12 months [111]. The AQoL-8D contains 35 items which load onto eight dimensions. Three of these dimensions are related to a physical ‘super-dimension’ and the remaining five to a psychosocial (‘mental’) super-dimension [111]. The AQoL-8D has been found to be valid and have good reliability compared to other multi-attribute utility instruments and is particularly suitable when evaluating economic outcomes where psychosocial elements of health are of importance [112].

#### Adverse and serious adverse events

To assess the efficacy of the intervention, adverse events will be recorded by the research team immediately upon their notification. An adverse event will be defined as ‘any untoward medical occurrence in a participant subject to the intervention’. A severe adverse event will be defined as ‘any event requiring hospitalisation or causing an inability to carry out usual activities’. In the occurrence of any adverse or serious adverse event, the research team will report to the governing ethics committee, review relevant risk assessments, aim to mitigate future risk of adverse events and provide the appropriate duty of care to the participant/s concerned.

### Data collection, management and monitoring

The principle investigator and trained research assistants will collect all data from the study participants. Inter- and intra-tester reliability will be determined for data-collecting investigators and research assistants for all outcome measures as appropriate. Computer files containing study data will be de-identified and password protected with access only available to study investigators. Data entry will be performed in duplicate and accuracy will be assessed with automatic cross-checking procedures. Range checks for data values will be performed to promote data quality. All hard-copy data files will be stored in a securely locked filing cabinet at the School of Human Movement and Nutrition Sciences, The University of Queensland. Disposal of hard-copy files will be performed in accordance with general procedures, the regulations of the administering ethical board and The University of Queensland.

Electronic copies of participant data and audio recordings and transcripts of peer supporter interviews will be stored in various modes (computer hard drive; external hard drive; and cloud-based systems) all of which will be de-identified and password protected, maintaining confidentiality at all times. Electronic data repositories will only be accessible by the study investigators and associated data analysts. Results will be made available to participants (including any publications that result from the project) upon individual request following the completion of the study. Individual participants will not be identified in any resultant manuscripts or reports of this trial.

The investigators will comply with the Good Clinical Practice (GCP) guidelines adopted by the Therapeutic Goods Administration and will document and appropriately address all adverse events through Human Research Ethics Committee of Bellberry Ltd. The study investigators will permit study-related monitoring, audits, and inspections by Bellberry Ltd. of all study related documents (e.g. source documents, regulatory documents, data collection instruments, study data) and study facilities (e.g. diagnostic laboratory). An independent data monitoring committee is not needed, given the role of Bellberry Ltd. This study will be conducted in full conformance with the principles of the ‘Declaration of Helsinki’ according to international standards of GCP guidelines, applicable Australian government regulations, and Institutional research policies and procedures.

### Following study completion

On completion of a participant’s enrolment in the study, they will be offered general exercise oncology recommendations and directed to nearby community exercise programs or Accredited Exercise Physiologists for continued care.

### Statistical considerations and data analysis

A priori power calculations determined that a sample of *n* = 188 (*n* = 94 per group) will be needed to detect a minimum between-group difference in MVPA levels at 12 months, with 80% power and 5% alpha. To account for an anticipated dropout rate of 20%, 226 participants (*n* = 113 per group) will thus be recruited for this study. A priori criteria for successful provision of peer support will be contacting participants on average on one or more occasions per week over the 12-month maintenance phase.

Data will be assessed for normality of distribution using the Kolmogorov-Smirnov test. Raw continuous data (e.g. minutes of MVPA per day) will be analysed using linear mixed (fixed and random) modeling, to assess changes over time and differences between groups. Group, time, and group × time interaction will be treated as fixed factors; participants will be treated as a random factor with individual intercepts. Each model will include covariates, as appropriate, and will be adjusted using the baseline value as a fixed continuous covariate as previously described by Courneya et al. [113]. Model residuals will be formally assessed for normality by use of the Kolmogorov-Smirnov test and visual inspection of histogram plots. Bonferroni pairwise adjustments will be made for all subsequent comparisons.

Non-continuous data will be analysed with the target distribution and relationship fixed to multinomial logistic regression. Statistical significance will be set at an alpha of *p* < .05 and analyses will be conducted using the intention-to-treat approach. There are no interim analyses planned or early trial termination guidelines. Data from the peer supporters will be analysed separately to those of the participants.

Qualitative data from the semi-structured interview transcripts will be assessed using inductive thematic analysis. Codes will be assigned to salient text segments across the entire data set, before codes will be combined to define overarching themes [108].

### Economic evaluation

A cost-utility analysis (CUA) from a societal perspective will be undertaken using patient level data collected alongside the clinical trial. A CUA compares the incremental costs and benefits, expressed in Quality Adjusted Life Years (QALYs) gained, of alternative programs. Costs will include direct costs (e.g. intervention cost) as well as indirect costs (e.g. lost productivity). Costs will be obtained by valuing the resources consumed using unit prices from standard costing resources such as the Medicare Benefits Schedule and relevant award wages in Australia. QALYs gained will be estimated, which is a measure of a person’s life expectancy, weighted by their utility score measured using the Assessment of Quality of Life – 8 dimensions (AQoL-8D) multi-attribute utility instrument at baseline, immediately following the supervised phase, and at 3, 6 and 12 months [111]. The incremental cost-effectiveness ratio (ICER) will be calculated, which is the difference in mean costs divided by the difference in mean QALYs. Sensitivity analysis will be conducted to assess the impact of input parameters (e.g. costs) on the results, and non-parametric bootstrapping will be used to characterise uncertainty around the ICER. In addition, sub-group cost-effectiveness analysis will be performed to explore heterogeneity of results between patients’ subgroups.

### Ethics and dissemination

This study has institutional ethical approval through the Human Research Ethics Committee of Bellberry Ltd. (#2015–12-840). Any future amendments to the protocol and/or associated documents will be submitted for approval through Bellberry Ltd.

Outcomes of this trial will be published in international, high-quality, peer-reviewed journals, and the findings will be presented at national and international conferences and meetings. Findings will also be communicated at community and consumer-led forums and will be presented at local hospital departments and university seminars.

## Discussion

The aim of the proposed trial is to determine the influence of peer support, compared to no peer support, on MVPA and various markers of health 12 months following a brief supervised exercise intervention in cancer survivors. If peer support significantly improves maintenance of MVPA compared to no peer support, it will emerge as a potentially sustainable and feasible means of enhancing cancer survivor’s engagement in physical activity following completion of a supervised exercise intervention. Further, should peer support also be shown to enhance the maintenance of physiological and psychosocial outcomes such as cardiorespiratory fitness, body composition and quality of life compared to no peer support, the long-term health and wellbeing of people living with and beyond cancer would be significantly improved. The economic evaluation will also provide evidence of the cost-effectiveness of peer support to maintain PA following a supervised exercise intervention.

Should peer support be shown to be (cost-) effective strategy to maintain MVPA following a supervised exercise intervention, it can potentially be adopted and implemented at a national level through existing peer support infrastructures of the State and Territory Cancer Councils, the primary providers of post-treatment support of cancer patients in Australia. A peer support-exercise model has the long-term potential to reduce comorbidities, improve physical and mental wellbeing, and significantly reduce the burden of disease in cancer survivors.

## Data Availability

Not applicable.

## Abbreviations

$\dot{\mathrm{V}}{\mathrm{O}}_{2\mathrm{peak}}$: Peak volume of oxygen consumption
AEP: Accredited Exercise Physiologist
AQoL-8D: Assessment of Quality of Life – 8 dimensions multi-attribute utility instrument
BMI: Body mass index
BSI: Brief Symptom Inventory
CCQ: Cancer Council Queensland
CRP: C-reactive protein
CUA: Cost utility analysis
DASS: Depression Anxiety Stress Scale
DXA: Dual energy x-ray absorptiometry
ELISAs: Enzyme-linked immunosorbent assays
EORTC QLQ-C30: European Organization for Research and Treatment of Cancer quality of life questionnaire for people with cancer
FACIT-F: The Functional Assessment of Chronic Illness Therapy: Fatigue
FACT-G: Functional Assessment of Cancer Therapy: General
GAD-7: Generalised Anxiety Disorder 7-item scale
GCP: Good Clinical Practice
HIIT: High intensity interval training
HR: Heart rate
HR_peak_: Peak heart rate
ICER: Incremental cost-effectiveness ratio
IGF: Insulin-like growth factor
IGFBP: Insulin-like growth factor binding protein
IL: Interleukin
ISAK: International Society for the Advancement of Kinathropometry
ISI: Insomnia Severity Index
MVPA: Moderate-to-vigorous physical activity
NHMRC: National Health and Medical Research Council
PA: Physical activity
PHQ-9: Patient Health Questionnaire-9
QALYs: Quality-adjusted life-years
RCT: Randomised controlled trial
SF-36: Short form 36 health survey
TNF-α: Tumour necrosis factor – alpha
WAI: Working Alliance Inventory
